# Nice to meet you, Law of Innovation...

**DOI:** 10.1016/S1808-8694(15)31190-3

**Published:** 2015-10-20

**Authors:** Henrique Olival Costa

In all kinds of endeavors in our life, we face moments when the crossroads on our way force us to take one path, leaving behind other options that did not seem to be the best ones. In general, although painful, this process represents a widening in the array of possibilities of the Endeavour and, mostly, a magnification of the potential prospects of our project. The Brazilian scientific community seems to be going through one of these moments. Created half a century ago, the national graduation system had in Newton Sucupira, an educational policy-writer during the military regime, not as much of a thinker as of a conceptualist. Strictly followed by the developers of Human Resources and Research fostering policies, in his words, the basis of the Graduation format can be found until today. We realize that the system’s main objective was to help the country reach its emancipation in knowledge generation, providing us with intellectual independence and releasing us from foreign dependence in what concerns Science and Technology.

The Graduation program gained momentum all over the world with the foundation of the Johns Hopkins University in 1876, especially created to develop Graduation studies and inspired on the idea of creative scholarship. In other words, a university created not only for the transmission of constituted knowledge, but also for the production of new knowledge through creative research activity (Infocapes99).

In fact, in Brazil, the analysis of by-laws and internal rules shows that, in general, schools fail to have an exact concept of the nature and objective of the Graduation, as its courses are frequently being confused with the specialization ones.

The Graduation program had its ups and downs, but the last five years have shown a pro-active interference policy in the purpose of programs under CAPES responsibility, stimulating programs and researchers with more scientific productivity, specifically by publishing their work in more visible and credible media (Figure 1). This led to a significant improvement in all Graduation-related aspects.

**Figure 1:**
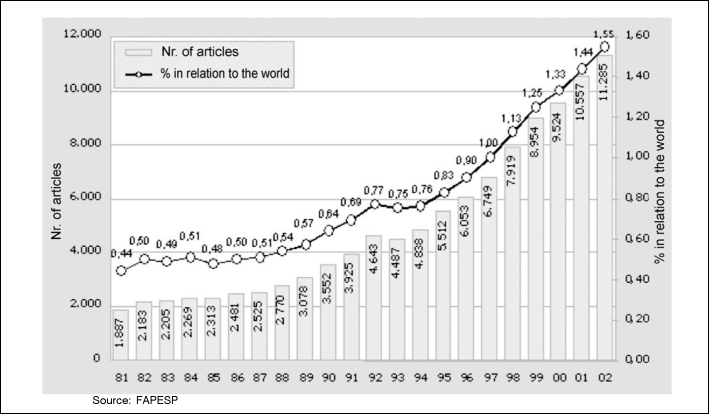
Brazil: Articles published in international scientific journals indexed in the Institute for Scientific Information (ISI) and percentage in relation to the world, 1981-2002

Source: FAPESP

Currently, the country has an extremely high numbers of Ph.D.s, around 8,000 in 2004, and scientific production continues to impressively rise. This tendency is clearer in the Health Area programs, which have surpassed all other knowledge areas in scientific production last year (Fig. 2).

**Figure 2:**
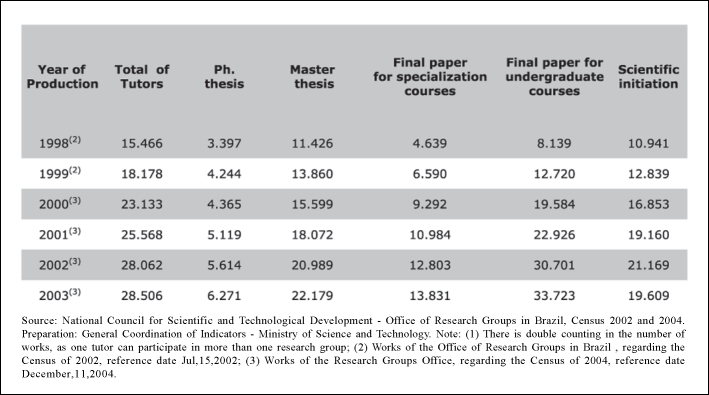
Brazil: Tutoring and concluded works according to the nature of the work, 1998-2003.

Source: National Council for Scientific and Technological Development - Office of Research Groups in Brazil, Census 2002 and 2004. Preparation: General Coordination of Indicators - Ministry of Science and Technology. Note: (1) There is double counting in the number of works, as one tutor can participate in more than one research group; (2) Works of the Office of Research Groups in Brazil , regarding the Census of 2002, reference date Jul,15,2002; (3) Works of the Research Groups Office, regarding the Census of 2004, reference date December,11,2004.

In the end, it seems that the system is succeeding to prepare, at least theoretically, an army of research technicians. If there is market and demand for their services, it is a question to be still answered.

Apparently, this group of professionals has not found their place in the productive society. And, even the thesis and articles published have been found more useful in the academic area than impacted the creation of products for life quality improvement.

One way of evaluating this impact is the number of patents generated in a society (Fig. 3).

**Figure 3:**
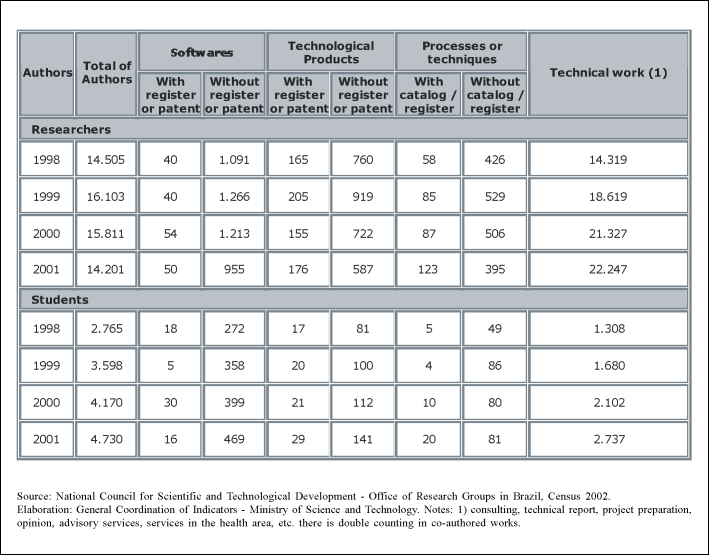
Brazil: Technical Production in the Research groups´ Office of the National Council for Scientific and Technological Development (CNPq), 1998-2001

Source: National Council for Scientific and Technological Development - Office of Research Groups in Brazil, Census 2002. Elaboration: General Coordination of Indicators - Ministry of Science and Technology. Notes: 1) consulting, technical report, project preparation, opinion, advisory services, services in the health area, etc. there is double counting in co-authored works.

As we can see, the quantity of registered patents in the country is much inferior to the number of graduated teachers, concluded thesis and published articles. Besides that, there is no indication of substantial increase or any increase of absolute number at all of patent applications in the Health area during past years.

If the initial purpose of the Graduation program was to provide the country with scientific autonomy, generating technological richness, in sum, intellectual property, we would understand that the last step of the journey is missing.

There we find our current crossroad. The implementation of Graduation programs has reached very high professional, conceptual and managerial status. Human Resources are being generated and production intensified, nevertheless, the prime purpose of the system suffers from lack of operational and financial instruments.

The operational problems have as an initial condition the fact that Graduation programs take place within the university, with all its academic hindrances and systematic inflexibility, while the richness production in Science and Technology is located at the productive sector, with its commercial and profitable purposes.

This binomial University-Company is the reason for a law at the Senate as Bill nr. 3.476/04 and that should be known by all of those involved with teaching and researching. Called Law of Innovation, it is about partnerships between companies, universities and scientific institutes aiming at a stronger partnership to stimulate the innovation process.

The need to improve the relationship between knowledge production and the business sector could be, at least in its largest part, considered by this project. Until today, given the difficulty of dialogue between university and company, Brazilian businesspeople end up choosing to import ready-to-use technology from abroad. As per the Law in the Senate, we might have news which may reflect, and if well used, determine significant changes in the chain of academic knowledge transmission to the population. The articles quoted below bring up situations that might be recognized by Teaching Institutions and Researchers as containing in its core unprecedented situations that can substantially modify their research procedure:

Art. 4. The Scientific and Technological Institutions (STI) may, by means of compensation and defined term:

I – share its laboratories, equipment, instruments, materials and other facilities with small companies in activities related to technological innovation to the accomplishment of incubation activities, without compromising its main activity;
II – allow the use of its laboratories, equipment, instruments, materials and other existing facilities by national companies and private non-profit organizations related to research activities, assuming that this permission does not directly interfere or conflict with its main activity;

Sole Paragraph. The permission and sharing mentioned at sub-paragraphs I and II of the **caput** will obey priorities, criteria and requisites established in the Edict approved by the highest body of STI, observing the respective availabilities and ensuring equal opportunities to interested companies and organizations.

Art. 5. The Federal Government and its entities are authorized to hold minority stake in a privately held company which serves a specific purpose, aimed at developing scientific and technological projects resulting in innovative products or processes.

Sole Paragraph. The intellectual property over the results achieved will belong proportionally to the institutions that hold stakes in the company.

Art. 9. STIs are allowed to make partnership agreements with private and public institutions to accomplish scientific and technological joint research activities and technology, product or process development.

§ 1º The public servant, military or officer of STI involved in the execution of the activities foreseen in the **caput**, may be granted a scholarship focused in fostering innovation, directly from a support institution or fostering agency.

Art. 10. The agreements and contracts signed between STI, support institutions, fostering agencies and private non-profit national entities related to research activities, whose purpose is compatible with this Law, may reserve resources for the coverage of administrative and operational expenses, observing the maximum limit defined by the rules.

Art. 13. To the creator, it is guaranteed a participation in profits received by STI, limited to one-third of the total. This profit must be a result of technology transfer contracts or protected creation exploitation, of which one has been the inventor, obtainer or author, applying, in whatever valid, to what is written in the Sole paragraph of art. 93 of Law 9.279, from May 14th 1996.

Art. 14. In order to duly execute what is presented in this Law, it is allowed to the public researcher to take a leave to collaborate in other STI, according to the terms of sub-paragraph II of the art. 93 of Law 8.112, of December 11^th^, 1990, noted the convenience of the original STI.

§ 2 During the leave period mentioned at the **caput**, it is guaranteed to the public researcher the salary of the effective position, military pay or salary of the public job of the original institution, besides the permanent pecuniary advantages determined by the law, as well as relevant and duly functional promotion and benefits of the social security plan.

Art. 15. At the discretion of the management of the public institution, the public researcher may be entitled to, provided that not on probation, an uncompensated leave to constitute a company aimed at entrepreneurial activity related to innovation.

## CHAPTER IV ON FOSTERING THE INNOVATION IN COMPANIES

Art. 19. The Federal Government, STIs and fostering agencies will promote and foster the development of innovative products and processes in national companies and private non-profit entities related to research activities, through grating financial, human, material or infra-structural resources to be adjusted through agreements or specific contracts, aimed at supporting research and development activities.

§ 1 Grating financial resources, under the format of economic support, financing or participation, aimed at the development of innovative products and processes, will be preceded by a project approval by the granting body or entity.

## CHAPTER V ON FOSTERING INDEPENDENT INVENTORS

Art. 22. To the independent inventor, who proves application for patents, it is allowed to ask for the adoption of their creation by STI, which will freely decide regarding convenience and opportunity presented by the request, aiming at the preparation of a project focused on its evaluation for future development, incubation, use or industrialization by the productive sector.

## CHAPTER VI ON INVESTMENT FUNDS

Art. 23. It is herein authorized to create Mutual Investment Funds in companies whose main activity is innovation, characterized by the communion of resources raised by means of distribution of securities, under Law 6.385, of December 7^th^, 1976, directed to the investment of securities issued by these companies to a variable portfolio.

The truth is the dice has been cast. According to the law, there are mechanisms that can facilitate the entry of funding for the academia through service provision, compensation increase or real profit making from knowledge production. There are pros and cons, the majority related to the supposed story of the industrial and the business sectors lack of disposition to invest in R&D, even though there are data available to contradict this belief.

According to FAPESP, public spending in research and development in the State of Sao Paulo between 1998 and 2002 was always above R$ 2.3 billion. Within this scenario, Sao Paulo is also the only State where State spending is higher than Federal spending: 60% to 40%, respectively, or R$ 1.47 billion compared to R$ 982 million in the four studied years. Another peculiarity about Sao Paulo refers to the participation of the business sector in R&D investments. In 2000, this participation reached 54%, or R$ 2.2 billion.

Data are there. The Bill has already been submitted through legal channels but still needs approval. It depends on everyone interested in Research to make good use of their prerogatives.

Regards,

